# Topological Engineering of Chiral Anomalies in Janus Nanoribbons

**DOI:** 10.1021/acs.nanolett.5c06221

**Published:** 2026-03-27

**Authors:** Vasil A. Saroka, Victor A. Demin, Michele Pizzochero

**Affiliations:** † Department of Physics, University of Rome Tor Vergata and INFN, Via della Ricerca Scientifica 1, 00133 Roma, Italy; ‡ Institute for Nuclear Problems, Belarusian State University, Bobruiskaya 11, 220006 Minsk, Belarus; ¶ Emanuel Institute of Biochemical Physics RAS, 4 Kosygin Street, Moscow 119334, Russia; § Department of Physics, 1555 University of Bath, Bath BA2 7AY, United Kingdom

**Keywords:** honeycomb lattice, graphene, topological insulators, topological order, flat bands, graph theory matchings

## Abstract

Topological band theory based on the variety of reciprocal space invariants provides an insightful framework for quantum material characterization in condensed matter physics. Its bulk–boundary correspondence principle has become a reliable tool for predicting Fermi level properties. Here we investigate a recently proposed bottom-up topological engineering based on reciprocal space invariants and show its connection to a real space *in situ* topology of a Kekulé structure originating in organic chemistry. We show that these approaches can be effectively used to engineer magnetic-field-free tunable chiral anomalies that are promising for energy-efficient electronic transport.

## Graphical abstract

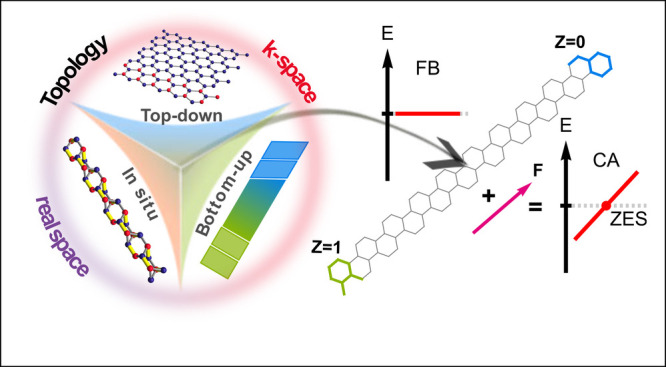

In the last two decades, topological band theory has established not only as a useful explanatory framework, but also as a practical tool for materials functionality engineering.^1^ Based on the *bulk–boundary correspondence* principle,^2^ which reliably predicts the electronic properties at the Fermi level, topological band theory allows one to design materials with target magnetic and transport properties. The robustness of such properties, due to nonlocal topological protection, keeps promises for such highly desired features as disorder-resilient transport,^3,4^ energy-efficient spintronics,^5–7^ fault-tolerant quantum computing,^8^ and sensing.^9^

The topological classification of condensed matter is branched and complex.^2,10,11^ It includes fundamental (time-reversal, particle-hole, chiral) and space (mirror, inversion, etc.) symmetries. It also depends on the dimensionality of the system and the type of topological invariant. In principle, topological materials come in two flavors: strong and weak. The flavors are distinguished by the involvement of the Brillouin zone dimensions in a topological invariant definition. In strong topological materials, all *n* dimensions of the Brillouin zone, $\Omega_{k}^{n}$ , are involved in the integration of the topological invariant, $\nu =\int_{\Omega_{k}^{n}}...d^{n}k$ , which can be either an integer (ν ∈ Z = {0, 1, 2, ...}) or a binary number (ν ∈ $Z_{2}$ = {0, 1}). In weak topological materials, only *n* – *l* dimensions of the Brillouin zone contribute to the invariant: ν­(*k*_1_, ..., *k*_*l*_) = $\int_{\Omega_{k}^{n-l}}...d^{n-l}k$ . As a result, *n*-dimensional weak topological material can be viewed as an *l*-dimensional “stack” of strong topological materials of *n* – *l* dimensionality, where *l* is called a codimension. Although this classification largely applies to insulator materials, where the topological invariants are determined on the quantum mechanical Hilbert space of the occupied states gapped from the nonoccupied states, similar considerations are possible for metals, where one switches focus to the geometry and topology of the Fermi surface.^12–14^ Such topological metals are commonly referred to as Weyl semimetals.^15^ Both metallic and insulating topological materials under specific conditions, such as boundary formation or external magnetic field application, can exhibit characteristics favorable for elastic backscattering-free transport. This feature can be theoretically described in the low-energy limit (continuum model) by the *chiral anomaly* of quantum field theory, which is rooted in gauge invariance and the polarization properties of the vacuum itself.^16–19^ In condensed matter, the chiral anomaly manifests itself as a linear mode of the electronic spectrum lacking a partner to accept the scattered electron,^20^ a situation taking place in various topological phases of matter, i.e., quantum Hall effects and Weyl semimetals, due to spin/valley locking or spatial separation of linear modes.^21,22^

Zero-energy modes of carbon nanostructures have been a subject of interest for a long time.^23–30^ Graphene nanoribbons (GNRs) provide a versatile platform for probing and advancing topological engineering of such modes.^31–36^ With the emergence of bottom-up self-assembling methods,^37^ it became possible to fabricate nanoribbons in an atomically precise fashion. This, in turn, has allowed the design of topological states in the bottom-up manner by engineering topological junction states at the interface of two GNRs and spatially translating the junction.^34^ This approach is qualitatively different from the “stacks” in weak topological insulators because it relies on real space rather than reciprocal space considerations. In fact, for the topologically protected one-dimensional bands reported in ref 34, there is no a two-dimensional system, which Brillouin zone could be fully or partially integrated to define a topological invariant to obtain the dispersive topologically protected bands via the bulk-boundary correspondence. Inspired by Song et al.,^36^ we further develop the bottom-up topological engineering of Rizzo et al.,^34^ wherein the topological unit cells of some narrow ribbon are combined in a finite length GNR to generate end states, which are then translated perpendicular to the translation vector of the narrow ribbon to obtain topological flat bands of a wide ribbon as shown in Scheme 1a. Hereafter, we refer to the narrow ribbon as a *base GNR* and to the wide ribbon as a *host GNR*. The width of the host GNR is equal to the length of the base GNR. As can be seen in Scheme 1a, the finite-length pieces of a base GNR with different end terminations commensurate with two unit cells of a base GNR are used as unit cells of a host GNR, i.e., as building blocks fused along their long terminations to obtain the host GNR. The geometry of the host GNR termination is determined by the unit cells of the base GNRs, while topological zero-energy modes originate from the base GNR unit cell topological invariants. Geometrically different unit cells chosen as end terminations of the base GNR give rise to the different termination geometries of a host GNR. The resulting ribbon with different geometries of the opposite terminations is termed a Janus nanoribbon. The flat bands of Janus ribbons are similar to those proposed for the generation of tunable chiral anomalies by means of an external electric field applied across the width of a GNR.^38–40^ Being motivated by this, we investigate whether this topological approach can provide a different perspective on the tunable anomalies.

**Scheme 1:**
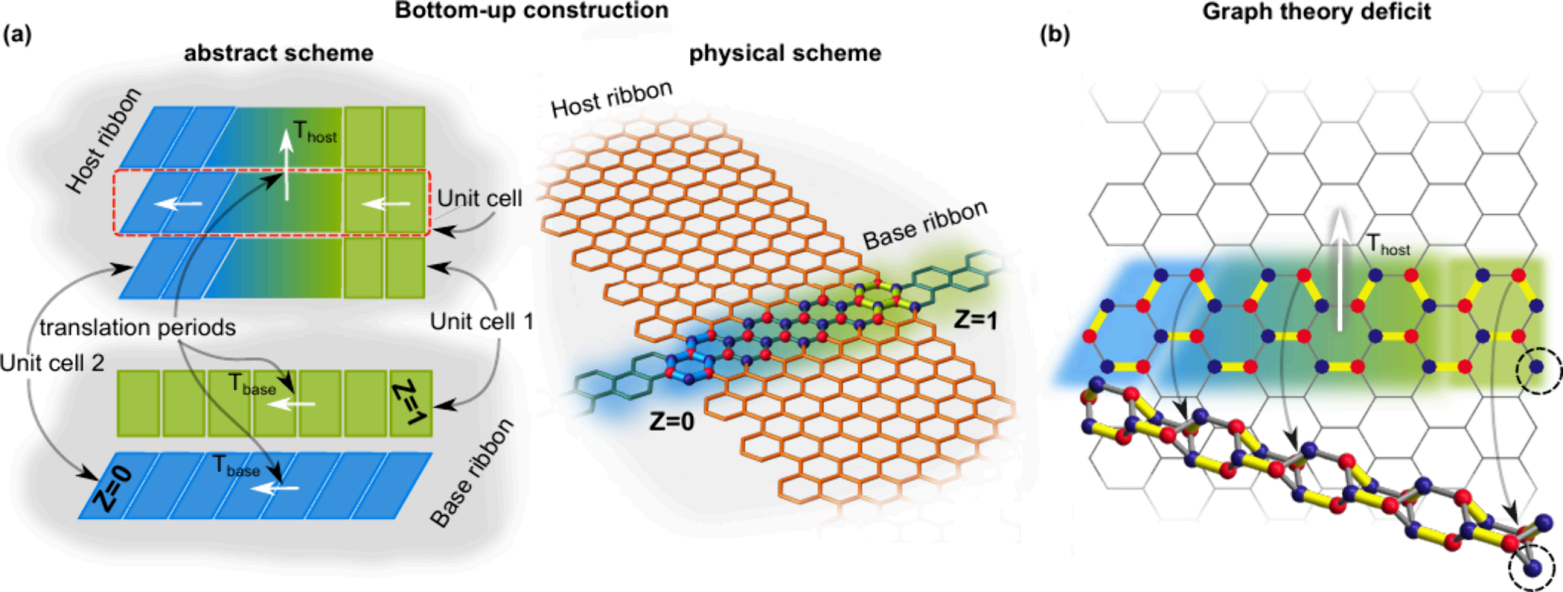
Two Equivalent Ways of Topological Engineering: (a) Bottom-up Topological Band Engineering; (b) Graph-Theoretic Deficit^a^

a For bottom-up topological band engineering (a), abstract and physical schemes are shown. White arrows show the key translations: *T*_host_ is the physical periodicity of the resulting host ribbon, while *T*_base_ is the translation used for the topological analysis of the base ribbon. Light green and light blue denote topologically non-trivial (Z≠0) and trivial (Z=0) unit cells of a base ribbon, respectively. The two sublattices of the honeycomb lattice, *A* and *B*, are colored in red and blue, respectively. The host ribbon is colored in orange, and the ghostly base ribbon is colored in green-blue. For the graph-theoretic deficit of the ribbon designed with bottom-up topological engineering (b), the host ribbon unit cell and its structural elements are highlighted using the color notations from (a). The non-adjacent edges—matchings—of the host ribbon unit cell graph are shown in yellow. A single vertex highlighted by a dashed circle represents the deficit η because it is not touched by any edge of the matching. The same red and blue colors as for honeycomb sublattices are used to distinguish disjoint vertex sets of the bipartite graph obtained by rolling up the host ribbon unit cell. The exact mapping between rolled-up graph vertices and the host ribbon lattice sites is shown by gray arrows.

In theoretical organic chemistry, the zero-energy states, which are characteristic for radicals, are related to the Kekulé patterns of the single and double bonds. When C=C double bonds cover all carbon atoms of an organic molecule, the Kekulé pattern arises. A single molecule can have many Kekulé patterns. However, when no such patterns are possible, then unpaired electrons appear leading to a radical formation.^41^ Such electron pairings resulting in zero-enegy nonbonding topological molecular orbitals can be described by the graph theory matchings and deficit.

In this Letter, we demonstrate that topologically engineered flat bands can be transformed into tunable chiral anomalies, thus delivering a unified variant of previous separate studies on electrical manipulations of Dirac fermions^42^ and flat band^43^ in GNRs. In what follows, we show how topological Z invariants^33^ can be related to quantum chemistry intuition based on covalent bond analysis that is connected to graph theory matchings and Kekulé structures.^41,44^ Then we screen the topological properties of a large number of simplified systems based on the nearest-neighbor tight-binding (TB) model with and without an external in-plane electric field to track tunable chiral anomalies. Finally, we propose a realistic system based on the termination of the nanoribbon with triarylmethyl radical^45^ promising for experimental realization of chiral anomalies via synthetic chemistry, as corroborated by our spin-polarized density functional theory (DFT) calculations.

## Topological Description

We begin by introducing topological properties at the nearest-neighbor TB level and graph-theoretic matchings for constructing flat bands with bottom-up topological approach. We then present the electronic properties within the TB model for the range of external fields to detect chiral anomalies in the flat-band structures. Neglecting a vanishing spin–orbit term,^7^ the TB Hamiltonian of π-electron network is

1 $$H=\underset{i}{\sum }\varepsilon c_{i}^{†}c_{i}+\underset{\left\langle i,j\right\rangle }{\sum }t_{1}\left(c_{i}^{†}c_{j}+c_{j}^{†}c_{i}\right)$$

where *c*_*i*_^†^ and *c*_*i*_ are electron creation and annihilation operators, respectively; ε is the on-site energy, which can be set to zero for all lattice sites without loss of generality (ε = 0); *∑*_⟨*i*,*j*⟩_ denotes summation over nearest neighbors; and *t*_1_ = 3.12 eV^46^ is the nearest-neighbor hopping integral. We note here that *t*_1_ varies for different structures,^47,48^ and therefore, we will use dimensionless units *E*/*t*_1_ in what follows. By going into reciprocal *k*-space in eq 1 via a Fourier transform, *c*_*i*_^†^ = $\frac{1}{\sqrt{N}}\underset{k}{\sum }e^{-ikR_{i}}c_{k}^{†}$ (and similarly for *c*_*i*_), where *N* is the number of unit cells and *R*_*i*_ is the site position, we solve the eigenproblem of the *n* × *n* matrix Hamiltonian *H*_*k*_ for a given unit cell of a GNR with *n* lattice sites in the cell. Then *k*-space Hamiltonian eigenvectors, *C*_*i*_(*k*) = (*C*_*i*,1_(*k*), *C*_*i*,2_(*k*), ..., *C*_*i*,*n*_(*k*))^T^, can be employed for the calculation of the Z invariant as^10,33^

2 $$Z=-\frac{i}{2\pi }\int_{1DBZ}Tr\left[q_{k}^{-1}\partial_{k}q_{k}\right]\;dk$$

where 1DBZ is the first Brillouin zone of a one-dimensional structure and *q*_*k*_ is the off-diagonal block of matrix *Q*:^49^

3 $$Q_{k}=1-2\overset{n/2}{\underset{i=1}{\sum }}C_{i}\left(k\right)⊗C_{i}^{†}\left(k\right)=\left(\begin{array}{ll} 0 & q_{k}\\ q_{k}^{†} & 0 \end{array}\right)$$

where *C* ⊗ *C* ^†^ is the outer product of a column vector with its Hermitian conjugate, yielding a matrix, and the summation ∑ runs over all the occupied states from 1 to *n*/2. *Q*_*k*_ originates from the fermionic projector formalism and for a chiral system can be viewed as *Q*_*k*_ = *f*(*H*_*k*_), where the function *f* maps positive (negative) eigenvalues of *H*_*k*_ to +1 (−1). The matrices *q*_*k*_ in eq 3 are *N*_*A*_ × *N*_*B*_ unitary matrices connecting the *A* and *B* sublattices of the honeycomb lattice within *Q*_*k*_, where *N*_*A*_ and *N*_*B*_ are the numbers of sites per sublattice. The integer Z tracks the phase winding of Det­(*q*_*k*_) across the 1D Brillouin zone. It can be interpreted as the difference between the Zak phases of the two sublattices.^33^ The chiral symmetry of *H*_*k*_ ensures that *Q*_*k*_ is representable through *q*_*k*_ as shown in eq 3. This results from the bipartite nature of the honeycomb lattice, which implies the absence of on-site energies in the bipartite tight-binding description (ε’s in eq 1). When a region with Z=p meets a region with Z=l , then |*p* – *l*| zero-energy modes (or flat bands) are expected at their interface according to the bulk–boundary correspondence principle.^2^

Graph-theoretically, the structure of a GNR can be seen as a simple bipartite graph, where lattice sites and bonds between them represent vertices and edges of the graph, respectively. Due to the translational symmetry of GNR such a graph can be seen as finite graph. That is, one can take one unit cell of the GNR and roll it along the translation period vector *T*_host_ as shown in Scheme 1b. This bipartite graph can be characterized in terms of collections of nonadjacent edges of the graph called matchings.^50,51^ The matching can be seen by chemists as the group of C=C double bonds within an organic molecule. Generally speaking, any simple graph can encounter the following three situations regarding matchings: (i) when the number of nonadjacent edges in the matching cannot be increased, then the matching is called *maximal matching*; (ii) when the number of nonadjacent edges in the matching is the largest possible for the given graph, then the matching is called *maximum matching*; and (iii) when the number of nonadjacent edges in the matching is the largest possible and covers all of the vertices of the graph, then the matching is called *perfect matching*. Perfect matching can be recognized by chemists as a Kekulé structure.

For the purpose of topological classification, we employ the number of vertices not covered by a maximum matching in the graph, called the *deficit* η = *n* – 2*c*, where *n* is the total number of vertices in the graph and *c* is the number of edges in the maximum matching. The graph deficit infer the radical behavior due to the absence of the Kekulé structures in the carbon nanostructure related to the graph. For any bipartite graph, i.e., featuring disconnected subsets of vertices, which are usually referred to as *A* and *B*, a maximum matching can be found using the Hopcroft–Karp–Karzanov algorithm.^52,53^ Thus, the deficit can be calculated using this algorithm. However, when the numbers of *A* and *B* vertices, *N*_*A*_ and *N*_*B*_, are different in the given bipartite graph, then the deficit η can also be calculated as the *sublattice imbalance* ^54^ or *color excess* ^55,56^ Δ = |*N*_*A*_ – *N*_*B*_|. The situation η = Δ > 0 is called class I topological frustration.^57^ It is also possible to encounter the case η > 0 with Δ = 0, which is called class II topological frustration.^57^ Using this classification, we define η = 0, corresponding to a perfect matching, as a trivial phase, while η > 0 as a topological phase. When η > 0, the absence of a perfect matching implies the presence of unpaired electrons leading to states that necessarily sit at the Fermi level, which coincides with zero energy in a bipartite, chiral-symmetric system. The defined topological phase holds a guaranteed number of zero-energy modes equal to η in accordance with the *nullity theorem* for bipartite graphs.^58^ Additional accidental modes may arise in general.

The effect of the external field applied to the flat band structures identified as described above is accounted for in eq 1 via on-site energies:^59^ ε = −*e* **F**·**r**, where *e* is the elementary charge, **F** is the homogeneous electric field strength, and **r** is the position of the lattice site. To visualize the localization of the electron wave function, in the energy band diagram we use a color scheme based on the inverse participation ratio (IPR):^60^ $IPR=\int_{V}{\left|\Psi \left(r\right)\right|}^{4}\;dr$ , where $\int_{V}{\left|\Psi \left(r\right)\right|}^{2}\;dr=1$ . For a normalized eigenvector *C*_*i*_(*k*), the IPR is

4 $${IPR}_{i}\left(k\right)=\overset{N}{\underset{j=1}{\sum }}{\left|C_{ij}\left(k\right)\right|}^{4}$$

For a perfectly localized state *i*, in which an electron density occupies only one atomic site, IPR_*i*_(*k*) = 1 (*loc*). However, IPR_*i*_(*k*) ∝ 1/*N* for any extended state, and this approaches zero in the thermodynamic limit: lim_*N*→*∞*_ IPR_*i*_(*k*) → 0 (*deloc*) (see the Supporting Data for code realization).

## Zigzag Crystallographic Direction

Figure 1 presents the results of the bottom-up flat band construction for host Janus GNRs with zigzag crystallographic orientation and translation period *T*_host_ = 2*a*, where *a* = 2.46 Å is the graphene lattice constant. Note that the case of *T*_host_ = *a* corresponds to the half-bearded GNR, whose chiral anomaly has been considered in detail in refs 38 and 39, and Z -invariant topological properties can be easily checked with an analytical formula for armchair GNRs from ref 33 applied to the base GNRs. The set of base GNR unit cells is given in Figure 1a together with the Z invariant for each of them. The common for all base unit cells band structure is shown in Figure 1b. A representative band structure of a host Janus GNR featuring flat in-gap modes (IGM) together with their wave functions is shown in Figure 1c. Similar results are given for an external field applied across the width of the GNR in Figure 1d. It is seen that the host Janus GNR with an even number of atoms in the unit cell can exhibit two flat bands. One of those transforms under an external field into a chiral anomaly with linear dispersion in each of the two valleys of the honeycomb lattice, **K** and **K′**, while another experiences a simple rigid shift. Investigation of other 20 combinations between unit cells from Figure 1a, shows no chiral anomalies for any trivial case $\left|\delta Z\right|=\left|Z_{\mathrm{left}}-Z_{\mathrm{right}}\right|=0$ , which confirms the topological origin of this peculiar chiral anomaly structure.

**Figure 1:**
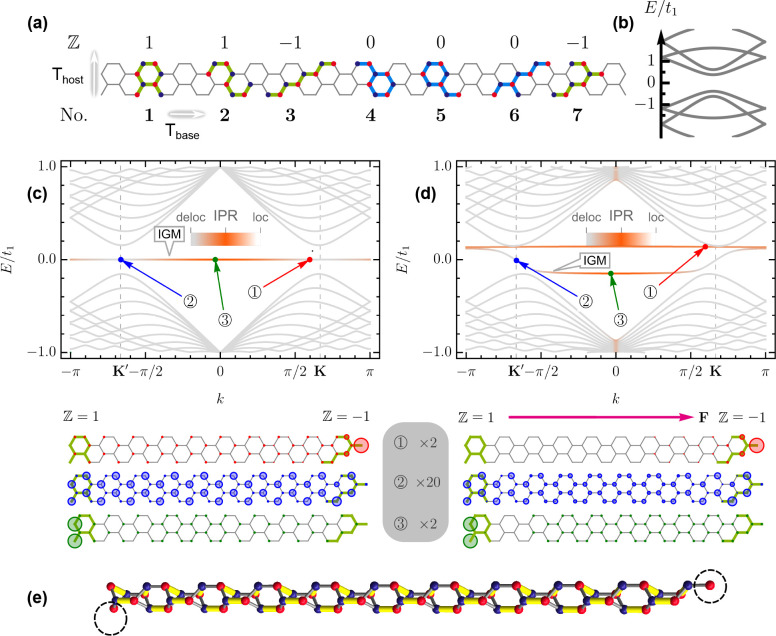
Bottom-up chiral anomaly engineering for Janus ribbons with zigzag crystallographic orientation. (a) Base armchair GNR with $T_{\mathrm{base}}=\sqrt{3}a$ for a host Janus ribbon with *T*_host_ = 2*a* (white arrows show translations *T*). Several topological (light green) and trivial (light blue) base GNR unit cells together with their Z invariants are shown. (b) Energy bands of the base GNR over the whole Brillouin zone. (c) Electronic bands and densities for three labeled states (below) for the Janus GNR with the *even* number of atoms in the unit cell and terminations defined by No. **1** and **7** base unit cells from (a). IPR, inverse participation ratio; IGM, in-gap mode. Due to high degeneracy of IGMs, an infinitesimal field can be added to the Hamiltonian in eq 1 to smoothly connect to the finite-field case. (d) Same as (c) but for the finite external field *eF*/*t*_1_ = 0.007 Å^–1^ (pink arrow). The gray legend with state numbers and their zoom factors is shared by (c) and (d). (e) Bipartite cylindrical graph of the host Janus GNR from (c, d). The maximum matching returned by the Hopcroft–Karp–Karzanov algorithm is shown in yellow. Dashed circles indicate vertices not covered by the maximum matching. *A* and *B* sublattices are depicted as red and blue in (a) and (e).

Table 1 summarizes results for host GNRs obtained from 21 randomly chosen combinations that cover Z=−1,0,1 . We observe that, despite the quite different nature of the Z invariant and graph deficit, they are always aligned in the given structures: |δZ|≡η . At the same time, we shall emphasize that the Z invariant carries slightly more information. Namely, it shall be noticed that the chiral anomaly structure is observed only for the ribbons where one of the base GNRs has Z=1 , whereas, for example, Z=−1 leads only to a rigid flat band shift in the external field similar to the shift seen for one of the flat bands in Figure 1d. As mentioned in ref 33, the sign of the Z invariant denotes the sublattice localization of the zero-energy mode. In addition, we notice that the sign of the difference of Z invariants from the opposite terminations is aligned with the sublattice imbalance Δ stripped of the modulus: $Z_{left}-Z_{right}$ = *N*_*A*_ – *N*_*B*_. Therefore, the chiral anomaly prefers one sublattice over another.

**Table 1:**
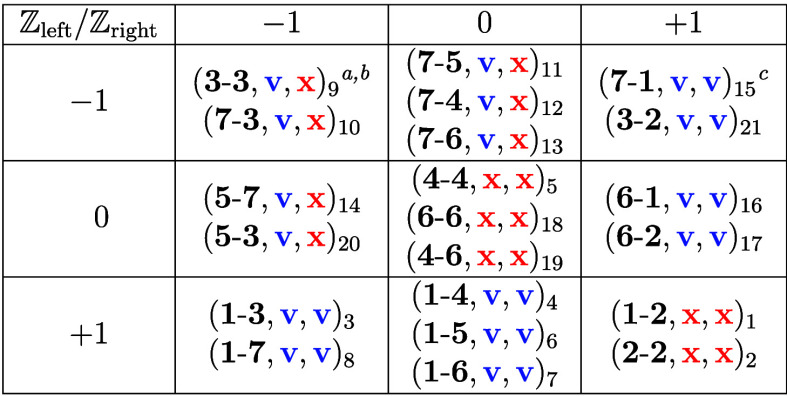
Atlas of Fully Flat Bands and Chiral Anomaly Linear Dispersions for GNRs with Zigzag Crystallographic Orientation and Translation Period *T*_host_ = 2*a*

a We use the triplet notation (**leuc**-**reuc**, fb, ld)_#_, where **leuc** is the left termination unit cell number as assigned in Figure 1a, **reuc** is the right termination unit cell number from Figure 1a, fb is the flag denoting fully flat band, ld is the flag denoting linear dispersion, and the triplet subscript # is the case study number. Flags can take the values **v** for yes and **x** for no.

b See band structures and details of the graph-theoretic analysis in the Supporting Data.

c This exceptional case is the same as case No. 8, where the GNR is rotated 180° around its translation symmetry axis. All other cases represent different GNRs.

## Armchair Crystallographic Direction

In Figure 2, we present how the Z invariant works for bottom-up topological engineering of host Janus GNRs with armchair terminations (see Supporting Note 1 that explains honeycomb lattice deformation opening a gap for gapless base ribbons while preserving chiral symmetry). For the host GNR with $T_{host}=\sqrt{3}a$ (cf. with *T*_host_ = *a* for zigzag host GNR), 8 unit cells are generally possible with Z ranging from −1 to 1, as can be seen in Figure 2a. Such unit cells are numbered with subscript index 1. A few analogous base GNR unit cells for a twice larger *T*_host_ are given in Figure 2b, where they are labeled with subscript index 2. Base GNR band structures featuring band gaps as explained above are shown in Figure 2c,d. The primary nontrivial configuration of Janus type is the one that combines the so-called twig termination^61^ with an armchair termination, and this is the example making a link between Ryu–Hatsugai loops^24^ (and their generalization by Delplace^29^) and our bottom-up topological engineering (see Supporting Note 2 and Figures S1 and S2). For this and all other configurations presented in Table 2, we find |δZ|≡η . To further verify |δZ|≡η equivalence, we consider host GNRs of armchair type with $T_{host}=2\sqrt{3}a$ (unit cells numbered with subscript index 2). These host GNRs admit more configurations of the base GNRs. Some of those are presented in Figure 2b. For these structures, we find a base GNR unit cell that is characterized by Z=2 , which justifies the use of Z instead of $Z_{2}$ in our topological description. The electronic properties of a host Janus ribbon, which features a base unit cell Z=2 , are presented in Figure 2e,f. As one can see from Figure 2g, in this peculiar case the graph-theoretic description predicts the fully flat bands equally well as the bottom-up topological construction.

**Figure 2:**
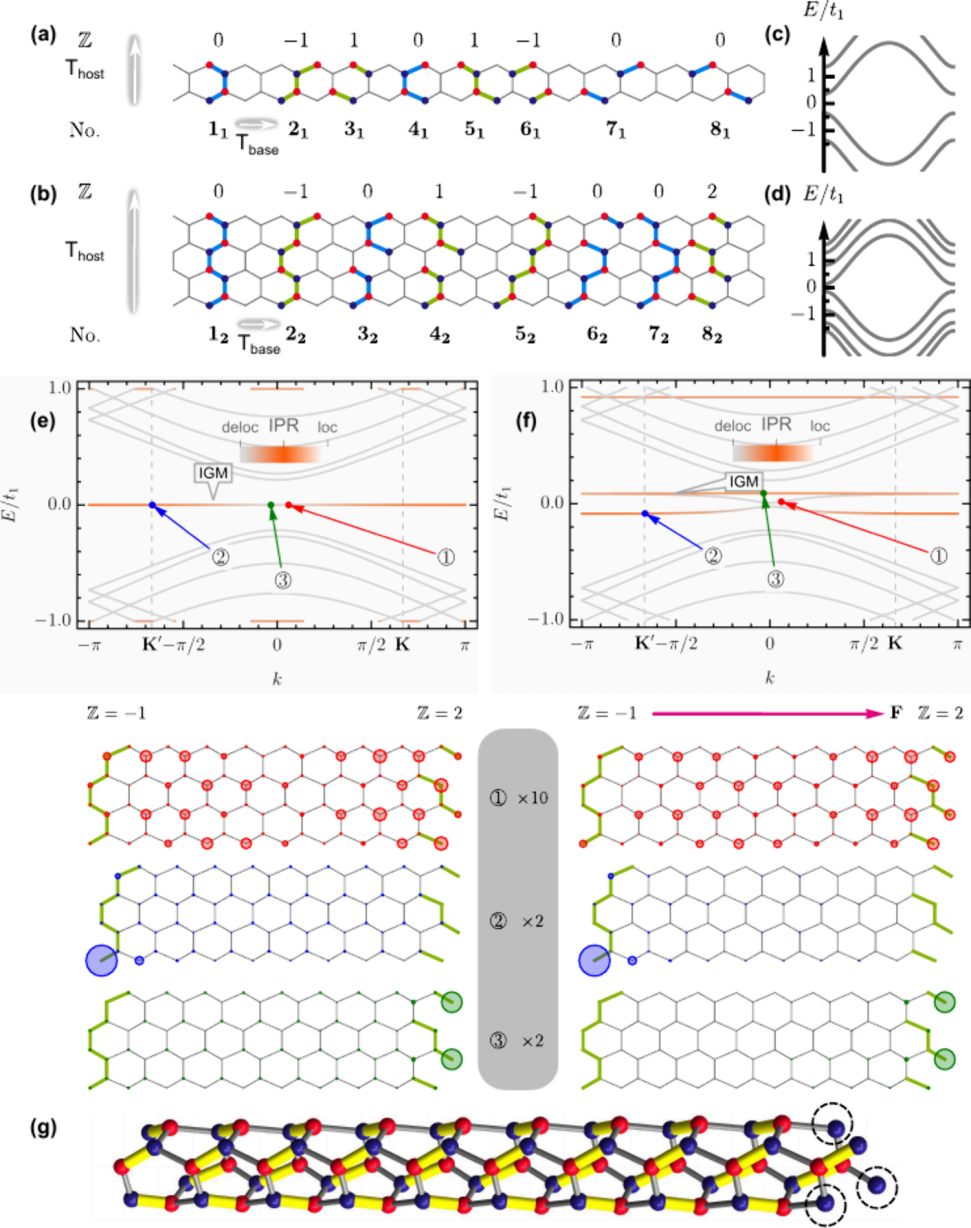
Bottom-up flat band engineering in host Janus GNRs with armchair orientation. (a) Base zigzag GNR with *T*_base_ = *a* for Janus GNRs with $T_{host}=\sqrt{3}a$ (white arrows show *T*’s). Several topological (light green) and trivial (light blue) base GNR unit cells with their Z values are shown. (b) Same as (a) but for $T_{host}=2\sqrt{3}a$ . (c, d) Energy bands for base GNRs in (a) and (b), respectively. (e) Electronic bands and densities for three labeled states (below) for a host Janus GNR with terminations **2**_**2**_ and **8**_**2**_ from (b). IPR, inverse participation ratio; IGM, in-gap mode. As in Figure 1c, here an infinitesimal field can be used to smoothly connect to (f). **K** and **K′** are the positions of the two valleys in zigzag GNRs, which for an armchair direction project into *k* = 0. (f) Same as (e) but for the finite external field *F* = 0.007 Å^–1^ (pink arrow). The gray legend with state numbers and their zoom factors is shared by (e) and (f). (g) The maximum matching for the host Janus ribbon is shown in yellow. Dashed circles indicate noncovered vertices. *A* and *B* sublattices are depicted as red and blue in (a), (b), and (g).

**Table 2:**
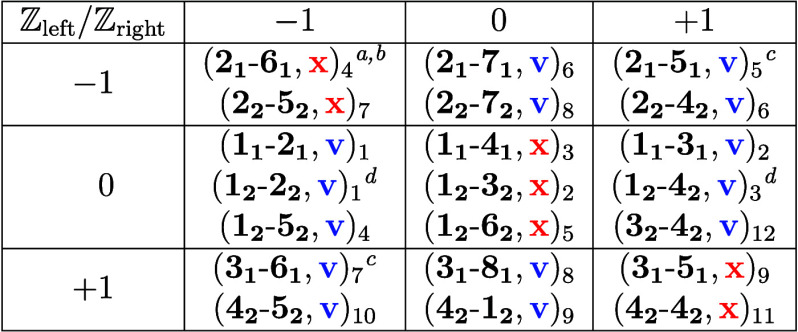
Atlas of Fully Flat Bands for GNRs with Armchair Crystallographic Orientation and Translation Periods $T_{host}=\sqrt{3}a$ (Subscript **1**) and $T_{host}=2\sqrt{3}a$ (Subscript **2**)

a We use a doublet notation (**leuc**-**reuc**, fb)_#_, where **leuc** is the left termination unit cell number as assigned in Figure 2a,b, **reuc** is the right termination unit cell number from Figure 2a,b, fb is the fully flat band flag, and the doublet subscript # is the case study number. Flags can take the values **v** for yes and **x** for no.

b See band structures and details of the graph-theoretic analysis in the Supporting Data.

c These are equivalent cases, where the host GNRs turn out to be the same.

d These are equivalent cases, where the host GNRs turn out to be the same.

Table 2 accumulates the results of 21 randomly chosen configurations of base GNR unit cells, covering Z=−1,0,1 . It is seen that flat bands can be reliably predicted by the Z invariant. In addition, the |δZ|≡η equivalence holds in all the cases.

Thus, we have seen that bottom-up engineering based on the topological invariant Z allows the construction of flat band materials. In contrast to some other termination-sensitive approaches,^24,29^ it cannot detect partially flat bands or some fully flat bands. However, those cases generally do not lead to the chiral anomalies and, in principle, can be considered as trivial or weakly topological flat bands as explained in Supporting Note 2.

## Synthetic Route to Chiral Anomaly

Although the peculiar terminations considered in the previous sections could be realized with metamaterials,^61–64^ they may be challenging for synthetic chemistry. We here thus propose a chemically feasible system for their realization. The termination presented in Figure 1c, holds similarity with a persistent triarylmethyl (TAM) radical that has been produced more than a century ago.^65^ In order to verify whether the termination embedment of such a radical, as schematically depicted in Figure 3a, can lead to the π-electron network and chiral anomaly, we perform spin-polarized DFT calculations,^66^ as implemented in the *SIESTA* package,^67^ see Supporting Note 3. The resulting optimized structure is given in Figure 3b.

**Figure 3:**
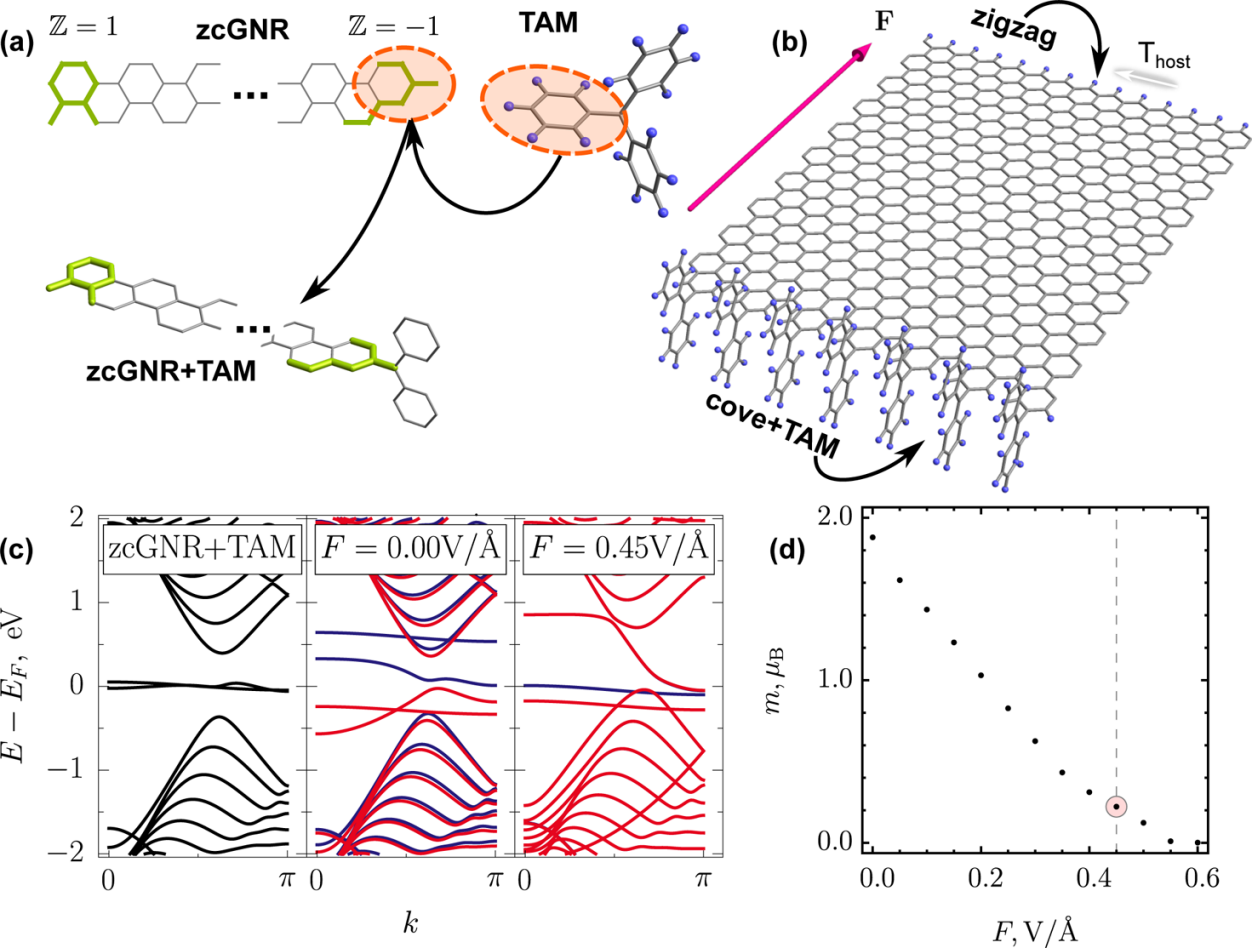
Chiral anomaly engineering with a trivalent carbon. (a) Schematics of the TAM functionalization of the termination of the host Janus ribbon from Figure 1: zigzag-cove GNR (zcGNR). Orange highlights the embedded benzene ring. Hydrogen (light blue) is shown only for the TAM radical. Other notations follow the conventions in previous figures. (b) Optimized geometry of the TAM-functionalized ribbon (7 unit cells are shown) together with *T*_host_ (white arrow) and the direction of the external in-plane field **F**, which has a reverse orientation with respect to the ribbon compared to Figure 1. Hydrogen is shown as light blue. (c) Electronic energy bands plotted over half of the Brillouin zone for TAM-functionalized zcGNR (left to right): nonmagnetic, spin-polarized without field, and spin-polarized in the external in-plane field *F*. Red and blue bands distinguish spin-up and spin-down species, respectively. (d) Unit cell magnetization (Bohr magnetons μ_B_) as a function of the field. The highlighted data point corresponds to the uttermost right band structure in (c).

In Figure 3c, we show that the spin-polarized DFT calculations support our hypothesis about the TAM performance. Such a ribbon exhibits the predicted topological flat bands, which spin split as a result of high density of states at the Fermi level, in qualitative agreement with Stoner’s criterion^68,69^ and quantitative agreement with Lieb’s theorem,^70^ but also demonstrates the electric-field-induced chiral anomaly. The widening of one of the two flat bands under the action of the field reduces the density of states, leading to a nonmagnetic transport channel, while the second flat band still supplies some magnetism, which also disappears at higher fields, as follows from Figure 3d and the Supporting Data. By changing the direction of the field it is possible to reverse the flat band behavior, as shown in Figure 1. We expect these results to persist even for the TAM-functionalized GNR on a substrate generating a termination strain from the nonplanarity of the TAM-functionalized termination, since the termination strain effects are generally small in GNRs.^71,72^

Finally, we note that all of the examples considered here featured sublattice imbalance Δ ≠ 0, and hence, η ≡ Δ and |δZ|≡Δ . However, a more general case of topological frustration is possible for GNR as for finite concealed non-Kekuléan hydrocarbons.^73^ Such topologically frustrated GNRs (at least few of them are known to us^73^) do not admit a bottom-up topological description in terms of Z invariant due to the peculiarity of their termination geometry. However, they do admit a description in terms of deficit η. The possibility of generating tunable chiral anomalies in such GNRs is a question under investigation. Thus, deficit can potentially describe flat bands in a wider range of nanoribbons. However, as we have seen above, the Z index sign can provide more information with respect to chiral anomalies in GNRs with the sublattice imbalance.

## Summary and Conclusions

We reveal an intricate connection between topological band theory in the form of a new bottom-up topological engineering and graph-theoretic real-space topology in the form of Kekulé structures: $\left|Z_{left}-Z_{right}\right|=\eta$ . This finding implies that bottom-up topological construction can be applied to zero-energy state engineering in finite nanotubes and nanobelts.^74,75^ The bottom-up topological engineering shows that tunable chiral anomalies can be observed in graphene nanoribbons lacking inversion symmetry but featuring an even number of atoms in their unit cell. However, this effect occurs only if $Z_{left/right}>0$ . A feasible synthetic chemistry route to the tunable chiral anomalies is offered by asymmetric triarylmethyl functionalization of the graphene nanoribbon terminations. An interesting future direction of investigation could be the possibility of generation of an internal electric field through asymmetric interface engineering, for example, with hexagonal boron nitride enabling fully planar systems^76,77^ or through functionalization with newly available trityl radicals.^78^ The impacts of the spin–orbit term and disorder would also be of great interest for prospective applications in spin-logic operations.

## Supplementary Material



## Data Availability

The data underlying this study are openly available in Zenodo at DOI:10.5281/zenodo.18997300.

## Notes

Details of the topological characterization of gapless base ribbons with Z invariant, top-down topological description of Janus ribbons in terms of Ryu–Hatsugai loops, and DFT methodology (PDF)
