# Supplementary material for: Topological Engineering of Chiral Anomalies in Janus Nanoribbons
Source: Nano Lett. 2026 Mar 27;26(20):6532–40. doi: 10.1021/acs.nanolett.5c06221 (PMC13220296; doi:10.1021/acs.nanolett.5c06221)
Supplement: Supplementary file 1 [file nl5c06221_si_001.pdf]

Supporting Information for Topological engineering of chiral anomalies in Janus nanoribbons

Vasil A. Saroka,*,†,‡ Victor A. Demin,¶ and Michele Pizzochero*,§

†*Department of Physics, University of Rome Tor Vergata and INFN, Via della Ricerca Scientifica 1, 00133 Roma, Italy*

‡*Institute for Nuclear Problems, Belarusian State University, Bobruiskaya 11, 220006 Minsk, Belarus*

¶*Emanuel Institute of Biochemical Physics RAS, 4 Kosygin Street, 119334 Moscow, Russia*

§*Department of Physics, University of Bath, Bath BA2 7AY, United Kingdom*

E-mail: vasil.saroka@roma2.infn.it; mp2834@bath.ac.uk

Supporting Note 1: The case of gapless base ribbons

In ref 1, it has been reported that the chiral anomaly cannot occur for Janus-type GNRs with zigzag orientation when $T_{\mathrm{host}} = 3a$. The next possible translation is $T_{\mathrm{host}} = 4a$. We remark that for such host GNRs, the base ribbons are amchair GNRs of the metallic family $n = 3p + 2$, where p is an integer. The degeneracy at the Fermi level leading to metallicity is lifted in realistic scenarious, i.e. accounting for long-range interactions or intrinsic strain[2–4] and these can be used to fix the topological nature of the base GNR as pointed out in ref 3. Similar ambiguity arises when one tries to apply this bottom-up approach engineering to host GNRs of armchair crystallographic orientation. Therefore, in order to corroborate the generality of the bottom-up topological principle, we investigate the ribbons with armchair crystallographic orientation. In this case, all base GNRs are zigzag, and they are all metallic

in the TB model for any width. Moreover, due to symmetry, the zigzag termination does not allow opening a gap by termination reconstruction.[2] The primary mechanism of band gap opening in zigzag GNRs is on-site electron-electron interaction, which is accounted for in the Hubbard mean-field model or can be emulated, to some extent, by an on-site staggered sublattice potential in the simple TB model. The on-site terms, however, break the chiral symmetry of the honeycomb lattice, thereby making $\mathbb{Z}$ invariant ill-defined. On the other hand, the $\mathbb{Z}_2$ invariant, introduced and used for topological classification of GNRs in refs 3,5, is insensitive to the on-site terms and, at first sight, could be a good alternative to $\mathbb{Z}$. However, $\mathbb{Z}_2$ is well-defined only if on-site terms in eq 1 of the main text preserve either mirror or inversion symmetry, which is a more restrictive condition, especially for Janus GNRs. The right way to proceed is to deform the honeycomb lattice anisotropically by introducing different t_1's to the three nearest neighbors. Varying this deformation does not affect the $\mathbb{Z}$ value as happens for $\mathbb{Z}_2$.[4] This opens a gap in the base GNRs with zigzag terminations and preserves chiral symmetry so that $\mathbb{Z}$ can be used for the topological description of host GNRs with armchair orientation.

Supporting Note 2: Top-down topological description of Janus ribbons

The purpose of this note is to link our bottom-up topological engineering to the top-down approaches known in the literature, in particular to the "weak" topological description in terms of parametric structures, which visualize the winding number or equivalently $\mathbb{Z}$ invariant protected by the chiral symmetry. In what follows, we refer to such structures as Ryu-Hatsugai loops.[6] A few comments shall be made before we proceed. Firstly, although there is a quite general description,[7] we focus on the basic geometries. Secondly, the generalized description in ref 7 deals with the Zak phase and, as such, must be attributed to the $\mathbb{Z}_2$ invariant. It seems that for an isotropic honeycomb lattice with 2×2 Hamiltonian

the situation is somewhat like for the 1D Su-Schrieffer-Heeger model,[8] i.e. the two definitions coincide and it is easy to mix and misname the two invariants. The parametric loops, however, clearly point on the $\mathbb{Z}$ invariant; therefore, we stick to this viewpoint.

Figure S1 presents the four basic GNR geometries for the honeycomb lattice and the derivation of the two periodic gauges for the graphene Hamiltonian. These two gauges give two possible phase portraits for the vector field in the reciprocal k-space as will be shown later. The geometries (1) zigzag, (2) bearded and (3) armchair have been considered in the seminal work.[6] The fourth twig geometry has been reported only recently.[9] It is important to note that Figure S1 presents the geometries for the ribbon that can be fully tiled by the base unit cells of the 2D honeycomb lattice. In other words, all of them admit perfect matchings, in effect, each base unit cell represents a matching, and therefore zero graph deficit η. In Figure S1, we also show the GNRs with mixed terminations. Those are incommensurable with the honeycomb lattice unit cell. They feature $\eta \neq 0$.

Figure S2 summarizes the results for the basic GNR terminations and their zero-energy modes on the honeycomb lattice. One shall notice that the two gauges identified in Fig S1 lead to two configurations of the vector field. Each such vector field can be parametrized by k_x and k_y, hence they lead to four possible parametric loops defined in the 3D-parameter space $(\mathrm{Re}[f], \mathrm{Im}[f], 0)$. The loops and their corresponding GNRs with the band structures are depicted below in Figure S2. It can be inferred from the band structures that zero-energy modes in zigzag and bearded geometries form a complementary pair that covers the full range of the Brillouin zone. The only difficulty is to resolve what happens at the Dirac point $k = 2\pi/3$, because in the wide-ribbon limit zero-energy modes may overlap at this point. To overcome this, we use the same approach as in Note 1 and introduce a small anisotropy to the lattice: $t_1 = 1.1t_2 = t_3$. As can be seen in Figure S2, this anisotropy shifts $k = 2\pi/3$ loops for zigzag and bearded GNRs in opposite directions with respect to the origin of the parametric space. Thus, the two zero-energy modes of the ribbons complement each other. A similar picture is observed for the GNRs with armchair and twig terminations.

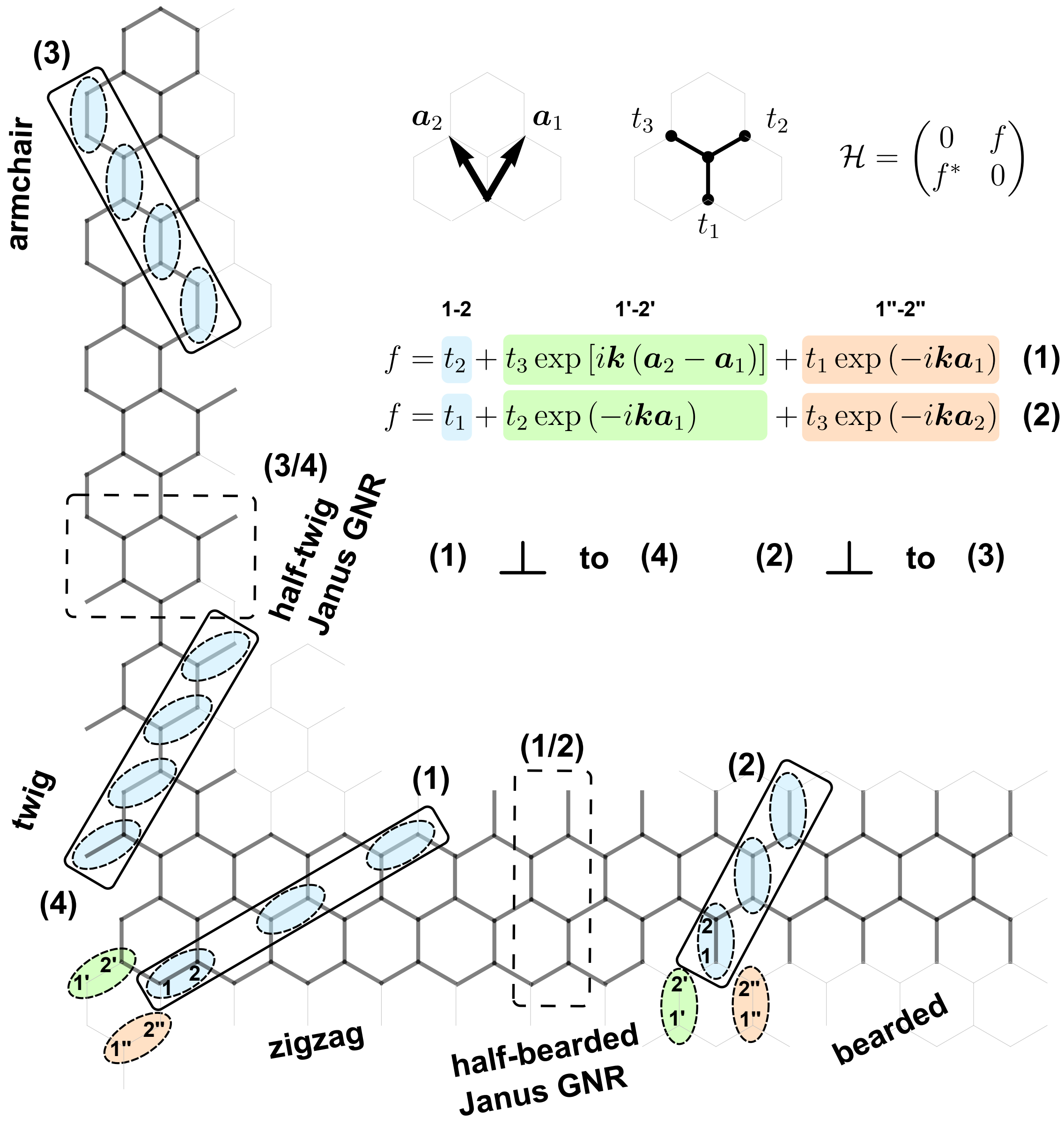

Figure S1. The GNR termination types (bold integer numbers) on a honeycomb lattice (thick gray) and the lattice tight-binding Hamiltonian $\mathcal{H}$ in the two possible periodic gauges. $\boldsymbol{a}_1$ and $\boldsymbol{a}_2$ are the primitive translations of the honeycomb lattice. $t_{1,2,3}$ are the hopping integrals for the three nearest neighbors. The base unit cells of the 2D lattice and their tillings resulting in the GNR unit cells are highlighted in light blue and dotted ovals. The neighboring unit cells of the 2D lattice contributing into the phase factor function f are highlighted with light green and light orange. The two atoms in the base and neighboring unit cells are enumerated with numbers and their primed versions, respectively. Janus ribbons unit cells each combining two basic termination geometries are shown by dashed boxes and labeled with bold fractions.

The loop $k = 0$ for the armchair GNR transforms from a line into an ellipse encompassing the origin of the parametric space (cf. with Figure 3c in ref 6), while similar loop for the twig termination ribbon shifts away from the origin.

The complementary pairs of terminations can be combined in the real space in a single Janus ribbon that forms either half-bearded GNR or a half-twig GNR, as can be seen in Figure S1. For such ribbons, the periodic gauge for the parametric loops cannot be smoothly defined as a function of parameter $k_{x,y}$ to describe zero-energy states. The only possibility to define loops through the whole Brillouin zone is to divide this zone into two regions corresponding the zero -energy modes of complementary terminations and to use the two different gauges accordingly. This situation is somewhat reminiscent of the Chern number tracing the obstruction with respect to the smooth gauge of the wavefunction on a 2D torus of the Brillouin zone.[10] This is a signature that Janus ribbons are linked to the "strong" topology.

It is also important to note that, by bottom-up engineering, all the ribbons in Figure S2 are trivial. Neither of those exhibits a fully flat band. However, the combination of complementary terminations in a single Janus GNR gives rise to a fully flat band and "strong" topology that is in direct equivalence with the real space *in situ* Kekulé patterns described by graph-theoretic deficit η as presented in the main text of the paper.

Supporting Note 3: Density functional theory calculations

In this study, we use the spin-polarized, gradient-corrected functional devised by Perdew, Burke, and Ernzerhof (PBE).[11] The Kohn-Sham orbitals of the valence electrons are represented as a linear combination of atom-centered basis functions of double-ζ plus single polarization (DZP) quality. Core electrons are replaced by norm-conserving pseudopotentials generated following the scheme proposed by Troullier and Martins.[12] A mesh-cutoff of

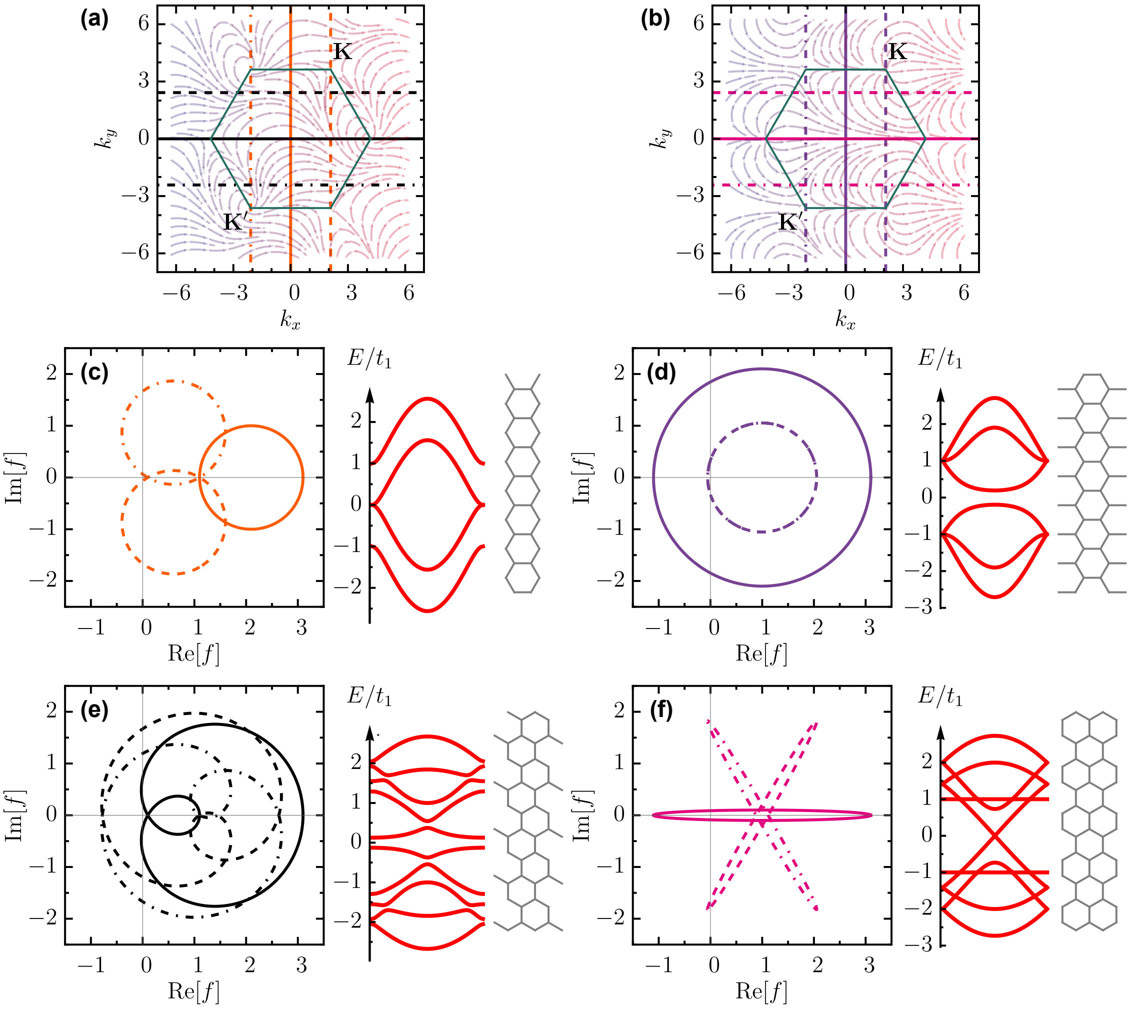

Figure S2. The top-down topological description of GNR terminations with Ryu-Hatsugai loops. (a, b) The vector field portraits corresponding to the two periodic gauges of f, i.e. (**1**) and (**2**) in Figure S1. (c, d, e, f) The winding loops in parametric space for $k = 0$ (solid), $2\pi/3$ (dashed), $-2\pi/3$ (dot-dashed) [up to $2/\sqrt{3}$ factor in armchair and twig cases]. $k \equiv k_x$ for zigzag and bearded GNRs, while $k \equiv k_y$ for twig and armchair GNRs. In each case, the loop parameter is a complementary one from (k_x, k_y) pair, which is changing along the lines of the corresponding style in (a) and (b). The band structures and their corresponding ribbons are shown to the left of each loop graphic. See Supporting Data in the main text for details of calculations.

400 Ry is used throughout. The integration over the Brillouin zone is carried out using a grid of 9 k-points along the periodic direction. Periodic replicas are separated by vacuum regions larger than 13 Å in the non-periodic directions. The atomic coordinates are optimized at zero field until the maximum component of the atomic forces is smaller than 0.02 eV/Å.

We note that, according to the Stoner criterion,[13,14] the expected flat bands at the Fermi level shall lead to the high density of states, and, therefore, the realistic system can be prone to ferromagnetic instability. Hence, we employ spin-polarized calculations.

References

(1) Saroka, V. A.; Kong, F.; Bogani, L.; Downing, C. A.; Payod, R. B.; Fischer, F. R.; Sun, X. Flat band, tunable chiral anomaly, and pitchfork bifurcation in a honeycomb lattice. *Phys. Rev. B* **2024**, *110*, 195134.

(2) Son, Y.-W.; Cohen, M. L.; Louie, S. G. Energy gaps in graphene nanoribbons. *Phys. Rev. Lett.* **2006**, *97*, 216803.

(3) Cao, T.; Zhao, F.; Louie, S. G. Topological phases in graphene nanoribbons: Junction states, spin centers, and quantum spin shains. *Phys. Rev. Lett.* **2017**, *119*, 076401.

(4) Tepliakov, N. V.; Lischner, J.; Kaxiras, E.; Mostofi, A. A.; Pizzochero, M. Unveiling and manipulating hidden symmetries in graphene nanoribbons. *Phys. Rev. Lett.* **2023**, *130*, 9045–9050.

(5) Lee, Y.-l.; Zhao, F.; Cao, T.; Ihm, J.; Louie, S. G. Topological phases in cove-edged and chevron graphene nanoribbons: Geometric structures, Z_2 invariants, and junction states. *Nano Lett.* **2018**, *18*, 7247–7253.

(6) Ryu, S.; Hatsugai, Y. Topological origin of zero-energy edge states in particle-hole symmetric systems. *Phys. Rev. Lett.* **2002**, *89*, 077002.

(7) Delplace, P.; Ullmo, D.; Montambaux, G. Zak phase and the existence of edge states in graphene. *Phys. Rev. B* **2011**, *84*, 195452.

(8) Cayssol, J.; Fuchs, J. N. Topological and geometrical aspects of band theory. *J. Phys. Mater.* **2021**, *4*, 034007.

(9) Xia, S.; Liang, Y.; Tang, L.; Song, D.; Xu, J.; Chen, Z. Photonic realization of a generic type of graphene edge states exhibiting topological flat band. *Phys. Rev. Lett.* **2023**, *131*, 013804.

(10) Benevig, B. A.; Hughes, T. L. *Topological Insulators and Topological Superconductors*, 2nd ed.; Princeton University Press: Princeton, New Jersey, 2013.

(11) Perdew, J. P.; Burke, K.; Ernzerhof, M. Generalized gradient approximation made simple. *Phys. Rev. Lett.* **1996**, *77*, 3865–3868.

(12) Troullier, N.; Martins, J. L. Efficient pseudopotentials for plane-wave calculations. *Phys. Rev. B* **1991**, *43*, 1993–2006.

(13) Stoner, E. C. Collective electron ferronmagnetism. *Proc. R. Soc. London. Ser. A. Math. Phys. Sci.* **1938**, *165*, 372–414.

(14) Stoner, E. C. Collective electron ferromagnetism II. Energy and specific heat. *Proc. R. Soc. London. Ser. A. Math. Phys. Sci.* **1939**, *169*, 339–371.
